# The influence of different sources of blood meals on the physiology of *Aedes aegypti* harboring *Wolbachiaw*Mel: mouse blood as an alternative for mosquito rearing

**DOI:** 10.1186/s13071-020-04465-9

**Published:** 2021-01-06

**Authors:** Luana Cristina Farnesi, Fabiano Duarte Carvalho, Anna Paula Canuto Lacerda, Luciano Andrade Moreira, Rafaela Vieira Bruno

**Affiliations:** 1 Laboratório de Biologia Molecular de Insetos, Instituto Oswaldo Cruz, Fiocruz, Rio de Janeiro, RJ Brazil; 2 Mosquitos Vetores: Endossimbiontes e Interação Patógeno-Vetor, Instituto René Rachou, Fiocruz, Belo Horizonte, MG Brazil; 3 Instituto Nacional de Ciência e Tecnologia em Entomologia Molecular (INCT-EM)/CNPq, Rio de Janeiro, Brazil

**Keywords:** *Aedes aegypti*, *Wolbachia*, *w*Mel strain, Blood feeding, Vector capacity

## Abstract

**Background:**

*Aedes aegypti* control programs have failed to restrain mosquito population expansion and, consequently, the spread of diseases such as dengue, Zika, and Chikungunya. *Wolbachia* infection of mosquitoes is a new and promising complementary tool for the control of arbovirus transmission. The use of *Wolbachia*-infected mosquitoes, mass reared using human blood, is currently being tested in several countries. However, the use of human blood for mass rearing mosquitoes, and thus expansion of this strategy, is problematic. With the aim of overcoming this problem, we tested the effect of different types of blood source on the fitness parameters of female* Ae. aegypti* and the *Wolbachia* titer over generations to be able to guarantee the suitability of an alternative source to human blood for mass rearing *Wolbachia*-infected mosquitoes.

**Methods:**

We investigated and compared essential parameters of the vector capacity of laboratory strains of *Ae. aegypti* with and without *Wolbachia* that fed on blood of different types of host (human, guinea pig, and mouse). The parameters analyzed were fecundity, fertility, pupation dynamics, and adult survival. Also, we tested whether it is possible to maintain mosquitoes with *Wolbachia* on mouse blood over generations without losing the bacterium titer.

**Results:**

The average number of eggs per female, egg viability and pupation dynamics in the *Wolbachia*-infected mosquito (*w*MelBr) strain were similar, regardless of the blood source. The F1 progenies of females that fed on mouse blood or human blood were analyzed. The longevity of males was lower than that of females. F1 female survival differed depending on the presence of *Wolbachia* in the mother. In subsequent generations analyzed up until F35, the relative *Wolbachia* density was even higher when mosquitoes fed on mouse blood in comparison to human blood.

**Conclusions:**

Taken together, our results provide no evidence that the different types of blood influenced the fitness of the *Wolbachia*-infected mosquitoes. The presence of the bacterium in the colonies of *Wolbachia*-infected *Ae. aegypti* after 35 generations under the conditions evaluated indicates that they can be maintained on mouse blood. Based on these results, we show that it is possible to use mouse blood to feed female mosquitoes when using human blood for this purpose is problematic.
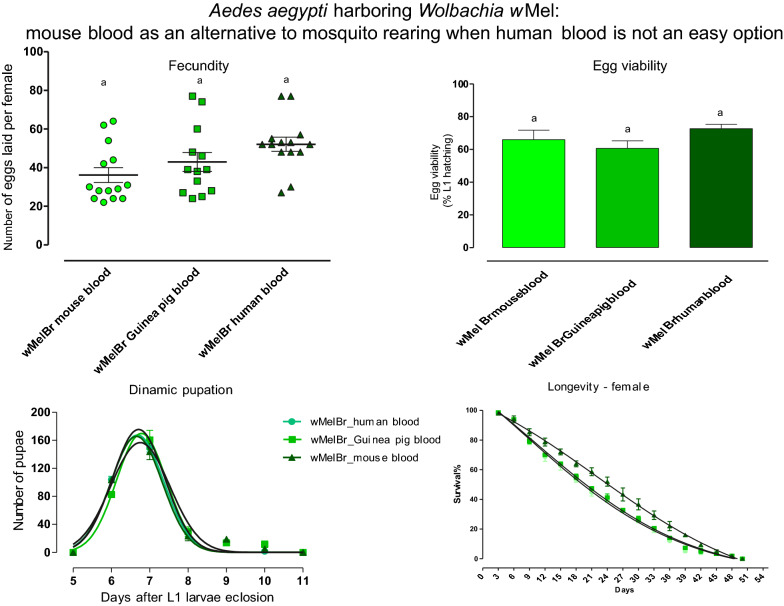

## Background

*Aedes aegypti*, a primary vector of arboviruses such as dengue, Zika, and chikungunya, has a wide geographical distribution and is found in tropical and subtropical regions around the world [1, 2]. Dengue, the main mosquito-borne arbovirus, is present in more than 100 countries, and there are an estimated 390 million cases of dengue infections in humans per year, according to the Pan American Health Organization [1]. A recent Zika virus pandemic had as its hallmark its rapid spread in the Western Hemisphere, where the vector *Ae. aegypti* is widely distributed [3]. Although first identified in the 1950s, there were only a few isolated cases of chikungunya virus before it reached the Americas in 2013; since then, it has spread throughout the world and become a major global concern [4].

Vector control programs have failed to restrain mosquito population expansion and, consequently, the spread of mosquito-borne diseases. One of the reasons for this is the increase of insecticide-resistant *Ae. aegypti* populations [5]. Furthermore, no specific treatment nor effective vaccines are yet available for these diseases [6].

A new complementary strategy to control arboviruses based on the endosymbiont *Wolbachia* has emerged, for which expectations are high. The bacterium *Wolbachia pipientis* is naturally present in individuals of most insect orders; however, it is not found in natural populations of *Ae. aegypti* [7, 8]. After a great deal of effort to artificially introduce *Wolbachia* into *Ae. aegypti*, it was found that the presence of certain strains of this bacterium strongly inhibit the replication of arboviruses, including dengue, Zika, and chikungunya [9, 10]. Due to a phenomenon called cytoplasmic incompatibility, reproduction leads to the rapid spread of *Wolbachia* through insect populations. Some countries have obtained approval for the release of mosquitoes infected with *Wolbachia* as a strategy to limit the transmission of arboviruses. Currently, this strategy is in the test phase in several countries, including Brazil [11].

Vector control programs, like the *Wolbachia* strategy, rely on mass rearing to enable the release of a substantial number of mosquitoes for the invasion of natural populations. *Ae aegypti* females are usually obligatorily anautogenous; they need a vertebrate blood meal for egg production, and prefer human blood for this [12, 13]. McMeniman et al. [14] showed that *Ae. aegypti* females infected with a virulent *Wolbachia* strain (*w*MelPop) were almost unable to produce viable eggs after a blood meal from non-human vertebrates such as chickens, guinea pigs, or mice (which are often used in laboratories for mosquito rearing), and only produced viable eggs when fed human blood. There is a considerable problem involved with the use of human blood for mosquito feeding: many countries do not have regulation to obtain volunteers that would accept to be bitten. Using blood from human donors, on the other hand, is also an obstacle to the mass rearing of mosquitoes. As a result, some studies have focused on the production of artificial diets for *Wolbachia*-infected mosquitoes to overcome the restrictions associated with them feeding solely on human blood. [15].

The aim of the present study was to test different kinds of laboratory animals on which *Ae. aegypti* harboring *Wolbachia* *w*Mel can feed to find a suitable source of blood that does not alter their fitness. Here, we analyzed the fitness parameters of *Ae. aegypti* infected with the *w*Mel strain when they fed on mouse, guinea pig, and human blood, compared with a natural population maintained under the same conditions, to establish an alternative blood source to human blood.

## Methods

### Mosquito lineages

Two lineages of *Ae. aegypti* without *Wolbachia* (Mosquitoes_Br and Rockefeller), which were considered as experimental controls for fitness in all of the assays, and one *Wolbachia*-harboring lineage (*w*MelBr), were used in the experiments. The Mosquitoes_Br strain was collected in the city of Rio de Janeiro, Brazil. To create the *w*MelBr lineage, males from the Mosquitoes_Br population were crossed, for several generations, with Australian female mosquitoes from the *Wolbachia*-harboring *w*Mel strain [5, 16]. Mosquitoes from the Rockefeller strain, an insecticide-susceptible reference lineage [17], were kindly provided by the Laboratório de Fisiologia e Controle de Artrópodes Vetores [Instituto Oswaldo Cruz (IOC), Fiocruz, Rio de Janeiro, RJ, Brazil].

### Mosquito maintenance

All assays were run under controlled laboratory conditions, as previously described [16]. Briefly, 300 mosquito larvae were raised at 25 ± 1 °C and fed with fish food (Marine granules; Tetra, Germany). Adult mosquitoes were kept in cages at 25 ± 2 °C and fed *ad libitum* with a 10% sucrose solution. Metamorphosis from the larval to adult stage was synchronized under a 12:12-h light:dark photoperiod, at constant 25 °C and 40–80% relative humidity, as described in Rezende et al. [18]. Blood meals required for egg production were performed on anesthetized guinea pigs, mice (CEUA-FIOCRUZ LW-20/14), or donated human blood using a Hemotek membrane feeder (Hemotek, Blackburn, UK). The blood used in all experiments of infective feeding was obtained from a blood bank (Hemominas) through an agreement signed between Fiocruz and Hemominas (OF.GPO/CCO-Nr224/16). Eggs on filter paper were hatched according to Farnesi et al. [19]. In all experiments, a random pool of mosquitoes was tested for the presence of *Wolbachia* by reverse transcription quantitative–polymerase chain reaction (RT-qPCR), as described below.

### Analysis of the efficiency of oviposition after *Ae. aegypti* fed on different blood sources

Five days after adult emergence, inseminated females were collected for blood feeding on various blood sources: guinea pigs, mice, or human blood bags, for 30–60 min. Following blood feeding, 20 females per experiment and blood condition were separated and distributed one-by-one among Petri dishes (150-mm diameter) lined with filter paper (Whatman no. 1). To each Petri dish, 4 mL filtered water was added to induce oviposition. After 1 h, the females were discarded, and the number of eggs per female was counted under a stereomicroscope (SteREO DiscoveryV.12; Zeiss). For each blood type, three independent experiments were performed. The methodology used to synchronize oviposition was adapted from Rezende et al. [18] and Vargas et al*.* [20].

### Analysis of egg viability

Egg viability was calculated as the hatching of L1 larva, adapted from Farnesi et al*.* [19]. Briefly, a total of 150 eggs per blood condition was placed in Petri dishes lined with a moist Whatman no. 1 filter paper. A 0.15% yeast extract solution was used as a hatching stimulus. Petri dishes with eggs were maintained in an incubator at a constant temperature (25 ± 1 °C). The relative humidity inside the incubator ranged from 40 to 80%. For each experiment, 150 eggs, randomly split into three independent triplicates (50 eggs each), were used. Three experiments were performed for each blood condition tested, i.e., 450 eggs were used in total.

### Evaluation of pupation dynamics and adult survival

The parameters were evaluated by simultaneously comparing Rockefeller, *w*MelBr, and Mosquitoes_Br strains reared under identical conditions of initial larval density and feeding, temperature, and photoperiod regimens. Eggs were induced to hatch for approximately 1 h. Three replicates of 300 newly hatched first-instar larvae were then randomly transferred to plastic trays (30 × 21 × 5 cm) with 1 L dechlorinated water and 0.15 g of fish food (Marine granules; Tetra). Fresh food was supplied every 3 days. The amount of food was sufficient for larval development.

#### Pupae formation

Pupation dynamics under the above conditions were examined daily as an indicator of cessation of larval development. This assay was performed three times.

#### Male and female longevity

The adults were randomly pooled in cylindrical cardboard cages (18 × 30 cm) 3 days after emergence and separated into groups of 50 couples, which received 10% sucrose solution ad libitum as the only food source. Mortality was scored every 2 or 3 days for approximately 2 months until all the infected mosquitoes had died. This assay was conducted three times.

### Selection of *Wolbachia*-infected *Ae. aegypti* fed on mouse blood after five and ten generations

Two fitness parameters were analyzed for F5 and F10 generations of *Ae. aegypti* (*w*MelBr) feeding on mouse blood: fecundity and egg fertility (following the same methodology used in the previous experiment). These data were compared with those of mosquitoes of the same strain that fed on human blood. *Wolbachia-*free mosquitoes of the Rockefeller lineage (Rock) were fed mouse blood and defined as an external experimental control.

### Evaluation of* Wolbachia* density over generations in *Ae. aegypti* fed on mouse blood

To ensure the presence of *Wolbachia* in the *Ae. aegypti* fed mouse blood throughout the generations, RT-qPCR was used for *Wolbachia* detection after 15, 20, 25, and 35 generations.

DNA extraction was performed by the squash buffer plus proteinase K method. After maceration of the mosquitoes in a Mini-BeadBeater (BioSpec, Bartlesville, USA) for 1.5 min, they were placed in a thermal cycler (Applied Biosystems, CA) at 56 °C for 5 min and then at 98 °C for 15 min. After this process, the samples were stored at − 20 °C.

RT-qPCR was performed using specific fluorescent probes in which the *Wolbachia w*Mel strain and the ribosomal protein of *Ae. aegypti* (RPS17S) were detected in the same reaction (duplex) (Table S1). RT-qPCR was performed using a LightCycler96 (Roche). The conditions were as follows: an initial step of 95 °C for 6 min, followed by 95 °C for 20 s, and 45 cycles at 95 °C for 3 s and 60 °C for 30 s and 72 °C for 1 s for fluorescence acquisition. The final volume of the reaction was 10 μL TaqMan Mix 4× (Applied Biosystems), 1 μM of each probe and primer, and 1 μL of each sample. Relative quantification was performed by comparing the cycle threshold values obtained for the* Wolbachia* surface protein* TM513* compared to the values of the 40S ribosomal protein S17 gene (*RPS17S*). Samples of *Wolbachia*-infected mosquitoes treated with an antibiotic (tetracycline) were used as a negative control.

### Statistical analysis

GraphPad Prism version 5.01 for Windows (GraphPad software, 1992–2007; GraphPad, San Diego, CA; www.graphpad.com) was used for all statistical analyses. For all experiments, mean and SD were calculated. One-way ANOVA followed by Tukey’s multiple comparison test, where *P* < 0.05 was considered statistically significant, was used in the fecundity and egg viability experiment. Linear regression analysis was performed for the analysis of pupation dynamics and survival.

## Results

### Efficiency of oviposition with different blood sources

**Fig. 1:**
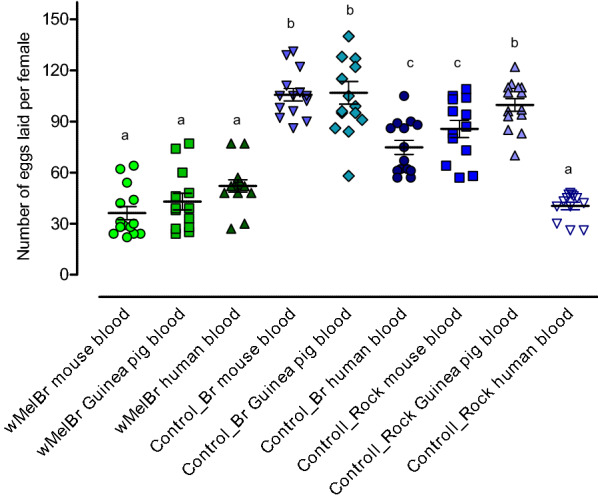
Eggs laid by individual female mosquitoes. In each experiment, we used five females per blood meal type. Three independent tests were performed for each type of blood meal.* Horizontal bars* represent the mean and SD.* Different letters* indicate significant difference according to one-way ANOVA followed by Tukey’s multiple comparison test (*F* = 38.83; *P* < 0.0001)

Figure 1 presents the overall female fecundity arising from various blood sources (guinea pig, mouse, and human blood bags) for all three lineages (Rockefeller, *w*MelBr, and Mosquitoes Br). *w*MelBr females laid fewer eggs than females without the bacterium (*P* < 0.0001). However, the average number of eggs laid per female *w*MelBr was the same regardless of the blood source (*P* < 0.0001). For mosquitoes without *Wolbachia*, in general, human blood lead to less egg production in comparison to the other blood sources.

### Egg viability

**Fig. 2:**
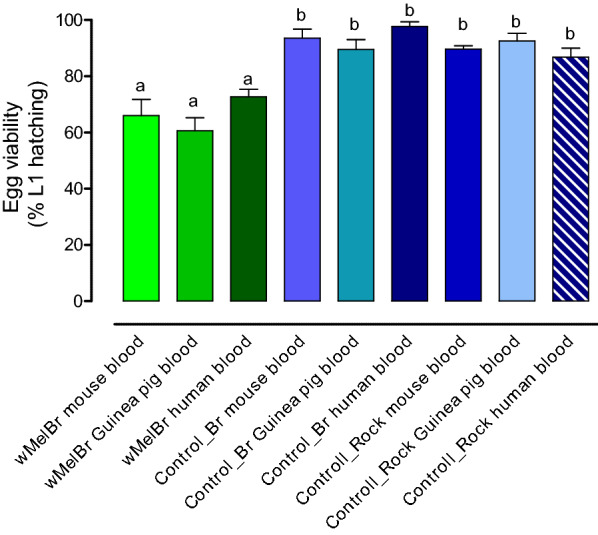
Egg viability was calculated as the percentage of hatched eggs of a random group for each blood meal condition. A total of 150 random eggs per blood condition was used in each experiment. Three independent experiments were performed for each type of blood meal (i.e., a total of 450 eggs per condition).* Bars* represent the SD.* Different letters* indicate significant difference according to one-way ANOVA followed by Tukey’s multiple comparison test (*F* = 13.65; *P* < 0.0001)

The egg viability was analyzed using a yeast solution as a hatching stimulus. In all lineages, with and without *Wolbachia*, there was no significant difference in the rate of viability after the mosquitoes fed on different blood sources (human, guinea pig, and mouse). On the other hand, the viability of *Wolbachia*-infected mosquitoes was, on average, lower (between 72.6 and 60.6%) than that of mosquitoes without the bacterium (between 97.6 and 89.6%) (Fig. 2).

### Pupation dynamics

**Fig. 3:**
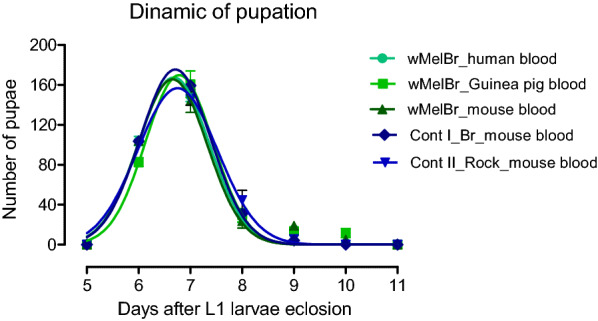
Pupation dynamics. A total of 300 larvae per blood condition was used in each experiment. Three independent experiments were performed for each type of blood meal (900 larvae per condition). For all conditions, the points were fitted to a Gaussian curve according to non-linear regression; *R*^2^ = 0.98, 0.97, 0.97, 0,98, 0.99, for the *Wolbachia*-infected strain ((*w*MelBr) fed human blood, *w*MelBr fed guinea pig blood, *w*MelBr fed mouse blood, ControlI_Br fed mouse blood, and Control II_Rockefeller fed mouse blood, respectively

When *w*MelBr, Rockefeller, and Mosquitoes_Br strains were reared under the same controlled conditions, the pupation dynamics were not significantly different. As expected, the majority of *Ae. aegypti* larvae pupated between 6 and 7 days after egg hatching (Fig. 3). The nonlinear regression analysis performed for all strains fitted a Gaussian curve (*R*^2^ = 0.98, *R*^2^ = 0.97, *R*^2^ = 0.97, *R*^2^ = 0.98, *R*^2^ = 0.99 for *w*MelBr_human blood, *w*MelBr_guinea pig blood, *w*MelBr_mouse blood, Rock_mouse blood, Mosquitoes_Br_mouse blood, respectively). There was no significant difference between the means (ANOVA followed by Tukey’s multiple comparison test; *F* = 4.766, *P* = 0.99).

### Comparative adult longevity of F1 progeny of* Wolbachia*-infected and non-infected females fed on different blood sources

**Fig. 4a, b:**
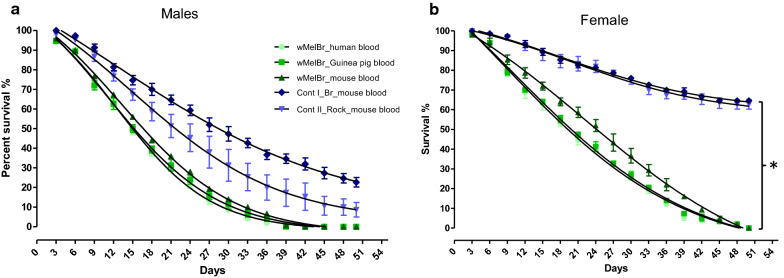
Longevity of progeny of females fed different sources of blood. Survival of *Ae. aegypti* infected with *w*MelBr (*green curves*) was compared with that of naturally uninfected and uninfected laboratory strains (*blue curves*). For each strain, three replicate groups of 25 male (**a**) or female (**b**) mosquitoes were maintained at 25 °C, 70–80% relative humidity, under a 12:12-h light:dark photoperiod. Three independent experiments were carried out in each case. There was no significant difference in male longevity according to one-way ANOVA followed by Tukey’s multiple comparison test (*F* = 2.4, *P* = 0.06). Regarding the females, there was no significant difference between the controls [*Wolbachia-*free mosquitoes of the Rockefeller lineage (*Rock*) × *w*MelBr] or among the *Wolbachia* strains. However, there was a significant difference between the progenies of infected mosquitoes and the controls according to one-way ANOVA followed by Tukey’s multiple comparison test (*F* = 10.57, *P* < 0.05)

To simplify the methodology, we decided to use mouse blood as the blood source for F0 control female without *Wolbachia*, i.e., Rockefeller and Mosquitoes_Br. We compared adult longevity (male and female) of the F1 progeny of females with and without *Wolbachia* that fed on mouse blood. For this assay, adults (both F1 males and females) were offered a 10% sucrose solution *ad libitum*. The results are shown in Fig. 4. As expected, male longevity was shorter than female longevity (cf. Fig. 4a, b). In F1 males, survival was not significantly different among all the tested conditions (ANOVA followed by Tukey’s multiple comparison test, *P* = 0.06).

The survival curves of F1 females of the control Rockefeller and *w*MelBr lineages were not significantly different (Fig. 4b) (ANOVA followed by Tukey’s multiple comparison test; *F* = 10.57, *P* > 0.05). The survival of F1 *Wolbachia* females after the mothers fed on any kind of blood was significantly lower than that of females of the bacterium-free strain (ANOVA followed Tukey’s multiple comparison test; *F* = 10.57, *P* < 0.05). The mortality of females without *Wolbachia* was only 30%, while it was 70% for females with *Wolbachia* after 30 days of observation (Fig. 4b). Taken together, our results did not provide any evidence that the type of blood affects the longevity of *Wolbachia-*infected mosquitoes.

### Selection of *Wolbachia*-infected *Ae. aegypti* fed on mouse blood after five and ten generations

We analyzed some essential parameters necessary to maintain a prolific *w*MelBr lineage in the laboratory, such as fecundity (number of eggs per female) and fertility (percentage of hatched eggs) after five and ten generations. The fecundity and fertility of *Wolbachia* (*w*MelBr) females when fed mouse blood were satisfactory. In general, after five and ten generations, fecundity and fertility were similar for all treatments (Table 1). These results show that it is possible to artificially select and maintain *Ae. aegypti* with the *Wolbachia w*Mel strain on mouse blood.

**Table 1:** Fecundity and viability of F5 and F10* Wolbachia*-infected *Aedes aegypti* (*w*MelBr) females fed mouse blood

Generation	Strain	Nulls (%)	Fecundity (%)	Fertility (%)
F5 (*n* = 30)	*w*MelBr (mouse)	16.6	84.6 (± 15.7) a	72.0% (± 28.5%) a
	*w*MelBr (human)	10	47.2(± 16.7) b	87.4% (± 18.5%) a
	*w*MelBr (human to mouse)	10	66.4 (± 19.7) a	78.6% (± 20.0%) a
	Control_Rock (mouse)	3	66.7 (± 9.9) a	84.6% (± 16.1%) a
F10 (*n* = 30)	*w*MelBr (mouse)	16.6	61.2 (± 37.6) a	77.5 (± 13.2) a
	*w*MelBr (human)	10	61.0(± 24.8) a	71.5% (± 17.5%) a
	*w*MelBr (human to mouse)	10	69.7(± 26.5) a	63.7% (± 11.5%) a
	Control_Rock (mouse)	3	127.3(± 27.6) b	75.5% (± 14.9) a

*Nulls* Percentage of females that did not lay eggs,* Fecundity* mean number of eggs per female,* Fertility* mean number of eggs hatched,* wMelBr Wolbachia*-infected strain,* Rock* *Wolbachia-*free mosquitoes of the Rockefeller lineage

*Different lowercase letters* represent statistically significant differences among strains in each generation (*P* < 0.05)

### Evaluation of* Wolbachia* density over generations in *Ae. aegypti* fed on mouse blood

**Fig. 5:**
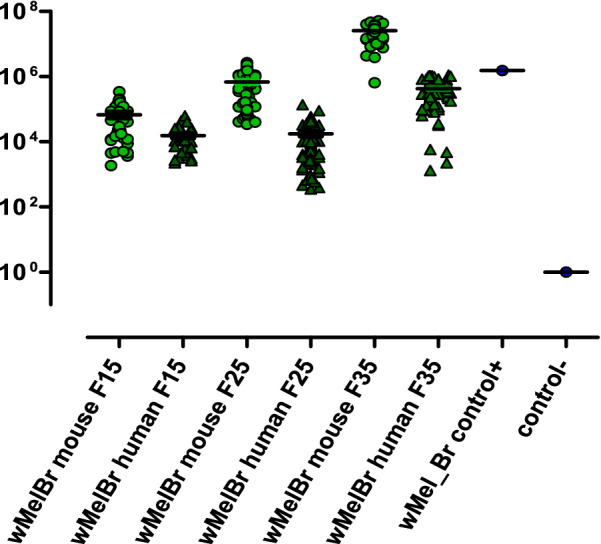
Relative Wolbachia density along with mosquito generations. The asterisks denote significant differences between generations F15, F25, and F35 (mouse blood × human blood) using *t*-Test following Mann Whitney test (*P* < 0.0001)

As it was possible to select *Ae. aegypti* with *Wolbachia* that fed on mouse blood and generated viable eggs, we decided to investigate whether this lineage would maintain its level of *Wolbachia* infection over many generations using only this blood source. In general, feeding on mouse blood did not have a detrimental effect on *Wolbachia* density in *Ae. aegypti* over the generations (Fig. 5). The presence of *Wolbachia* in the colonies of the *w*MelBr strain after 35 generations indicated that it is safe to feed the female mosquitoes on mouse blood. There was a significant difference between the F15, F25, and F35 (*P* < 0.05) generations according to the *t*-test following the Mann–Whitney test for pairwise comparisons (mouse blood × human blood). In all cases, the relative *Wolbachia* density of mosquitoes that fed on mouse blood was higher than that of mosquitoes kept under the same conditions but fed human blood.

## Discussion

*Aedes aegypti* is responsible for the transmission of many arboviruses worldwide, to many of which there are no specific antivirals or vaccines available [21, 22]. To overcome issues related to the massive use of insecticides, alternative and innovative approaches to the control of mosquito populations are currently being tested and evaluated, such as the use of bacteria of the genus *Wolbachia* [23–25]. Methodology based on the field release of *Ae. aegypti* infected with *Wolbachia* for dissemination amongst wild mosquito populations has been successfully tested by the World Mosquito Program [11] in many countries, e.g., Brazil, Colombia, Mexico, and Vietnam [27]. This strategy requires the large-scale dissemination of *Wolbachia*-infected mosquitoes, and the mass production of infected mosquitoes is mainly dependent on blood feeding. Laboratory strains of *Ae. aegypti* are generally able to withstand lab conditions when feeding on the blood of a wide variety of non-human vertebrates, including guinea pigs, chickens, mice, and sheep [17, 28]. However, feeding by a virulent strain (*w*MelPop-infected mosquitoes) on non-human animal blood sources (mouse, guinea pig, or chicken) resulted in the loss of reproductive capacity, reduced fecundity and hatching rate [14]. McMeniman et al. [14] showed that there is an interaction between the host blood and egg development in *w*MelPop-infected *Ae. aegypti* [14]. Based on this finding, the use of standard laboratory animal blood (chicken, mouse, guinea pig and sheep) was not considered suitable for feeding mosquitoes carrying the *w*MelPop strain. Thus, because of the proven higher levels of egg viability and other physiological parameters analyzed in mosquitoes carrying the *w*MelPop strain, the Word Mosquito Program currently uses human blood to this end [11, 13].

*Ae. aegypti* infected with the *w*Mel strain is highly refractory against dengue, Zika, chikungunya, and Mayaro [5, 9, 10, 26, 27]. The use of this mosquito for population replacement strategies requires its stable mass production. In this study, we aimed to overcome the necessity for human blood for mass rearing *w*Mel mosquitoes. In addition to human blood, we tested the blood of guinea pigs and mice for mosquito feeding and assayed and compared some physiological parameters.

Our first experiment compared the fecundity of females using guinea pig and mouse as blood sources in comparison with human blood. Overall, the fecundity decreased significantly in *Wolbachia*-infected mosquitoes compared to mosquitoes without the bacterium. However, in females with *Wolbachia* (*w*Mel), the number of eggs laid did not statistically differ, regardless of the blood source (Fig. 1). Using the same strain of *Wolbachia*-infected mosquitoes (*w*Mel), Dutra et al. [15] observed a similar average fecundity when they fed the females with human blood. On the other hand, Paris et al. [29] found that the fecundity of *w*Mel mosquitoes fed on pig or sheep blood decreased, although not significantly, compared to that of mosquitoes that fed on human blood. Taken together, these data suggest that it is not only the bacterial strain that affects fecundity when the blood meal comprises non-human blood, but also the type of host vertebrate used for the mosquito blood meal. Our data showed that egg viability was impaired when *Wolbachia* was present; however, there was no significant difference among the three sources of blood (Fig. 2). Paris et al. [29] showed that the viability of eggs of females infected with *w*Mel that fed on sheep or pig blood was impaired. Egg viability is more critical than fecundity to guarantee the perpetuation of *Wolbachia*-infected mosquitoes in the field.

Some parameters can influence larval development time and pupae formation in *Aedes*, such as temperature, nutrient supply, and insect growth regulators [30, 31]. Interestingly, in our assays, neither *Wolbachia* presence nor the blood source offered to parent mosquitoes affected the development time of immatures, since the pupation dynamics were not significantly different (Fig. 3). Under the conditions used here, the majority of pupae emerged between days 6 and 7 after egg hatching, as expected for this species when larval breeding temperatures are between 24 and 28 °C [30, 31].

In all analyses of longevity, male survival was lower than that of females, as expected for *Ae. aegypti* according to studies carried out under similar laboratory conditions [32, 33]. In general, the *w*MelBr mosquitoes had lower survival rates than the naturally uninfected and uninfected laboratory strains. However, compared with the *w*MelPop strain [33], the life span of *w*Mel was much improved. On the other hand, it is important to note there was no evidence that the type of blood influenced adult longevity (Fig. 4).

Paris et al. [29] found that *w*Mel-infected *Ae. aegypti* raised on non-human blood sources exhibited reduced *Wolbachia* density compared to when they were raised on human blood. *Wolbachia* density of a mosquito could influence transmission blocking of arboviruses such as Zika, dengue and chikungunya. If low *Wolbachia* densities are inherited over generations, the use of *Wolbachia* infection as a strategy to reduce the transmission of arboviruses could become ineffective [34]. Due to their diverse and overlapping host-feeding patterns, important mosquito vectors such as *Culex pipiens* and *Ae. aegypti* can be kept under laboratory conditions with different sources of blood. Further studies should be carried out to examine the fitness of mosquitoes fed different kinds of host blood as a means of increasing the size of laboratory colonies of these vectors [35].

We were able to overcome problems associated with the use of human blood for mosquito rearing by using mouse blood to feed females, and proved, for the first time, that it is possible to maintain strains of *Ae. aegypti* infected with *w*Mel on this type of blood without affecting the density of the bacterium over generations and without significant fitness costs to the mosquitoes.

## Data Availability

Data supporting the conclusions of this article are included in the article. All the datasets used in this manuscript are available for consultation from the corresponding author upon request, and can be accessed at the Laboratório de Biologia Molecular de Insetos (IOC-FIOCRUZ).

## References

[1] Bhatt S, Gething PW, Brady OJ, Messina JP, Farlow AW, Moyes CL (2013). The global distribution and burden of dengue. Nature..

[2] Hahn MB, Eisen RJ, Eisen L, Boegler KA, Moore CG, McAllister J (2016). Reported distribution of *Aedes* (*Stegomyia*) *aegypti* and *Aedes* (*Stegomyia*) *albopictus* in the United States, 1995–2016. J Med Entomol..

[3] Patterson J, Sammon M, Garg M (2016). Dengue, Zika and chikungunya: emerging arboviruses in the New World. West J Emerg Med.

[4] Nunes MR, Faria NR, de Vasconcelos JM, Golding N, Kraemer MU, de Oliveira LF (2015). Emergence and potential for spread of chikungunya virus in Brazil. BMC Med..

[5] Garcia GA, Sylvestre G, Aguiar R, da Costa GB, Martins AJ, Lima JBP (2019). Matching the genetics of released and local *Aedes aegypti* populations is critical to assure *Wolbachia* invasion. PLoS Negl Trop Dis..

[6] Pang T, Mak TK, Gubler DJ (2017). Prevention and control of dengue-the light at the end of the tunnel. Lancet Infect Dis..

[7] Zug R, Hammerstein P (2012). Still a host of hosts for *Wolbachia*: analysis of recent data suggests that 40% of terrestrial arthropod species are infected. PLoS ONE.

[8] Oliveira CD, Gonçalves DS, Baton LA, Shimabukuro PHF, Carvalho FD, Moreira LA (2015). Broader prevalence of *Wolbachia* in insects including potential human disease vectors. Bull Entomol Res..

[9] Moreira LA, Iturbe-Ormaetxe I, Jeffery JA, Lu G, Pyke AT, Hedges LM (2009). A *Wolbachia* symbiont in *Aedes aegypti* limits infection with dengue, chikungunya, and* Plasmodium*. Cell..

[10] Dutra HL, Rocha MN, Dias FB, Mansur SB, Caragata EP, Moreira LA (2016). *Wolbachia* blocks currently circulating Zika virus isolates in Brazilian *Aedes aegypti* mosquitoes. Cell Host Microbe..

[11] World Mosquito Program. Database. http:// https://www.worldmosquitoprogram.org/. Assessed 06 Oct 2020.

[12] Clements AN (1992). The biology of mosquitoes: development, nutrition and reproduction.

[13] Attardo GM, Hansen IA, Raikhel AS (2005). Nutritional regulation of vitellogenesis in mosquitoes: implications for anautogeny. Insect Biochem Mol Biol..

[14] McMeniman CJ, Hughes GL, O'Neill SL (2011). A *Wolbachia* symbiont in *Aedes aegypti* disrupts mosquito egg development to a greater extent when mosquitoes feed on nonhuman versus human blood. J Med Entomol..

[15] Dutra HLC, Rodrigues SL, Mansur SB, de Oliveira SP, Caragata EP, Moreira LA (2017). Development and physiological effects of an artificial diet for *Wolbachia*-infected *Aedes aegypti*. Sci. Rep..

[16] Farnesi LC, Belinato TA, Gesto JSM, Martins AJ, Bruno RV, Moreira LA (2019). Embryonic development and egg viability of* w*Mel-infected *Aedes aegypti*. Parasites Vectors.

[17] Kuno G (2010). Early history of laboratory breeding of *Aedes aegypti* (Diptera: Culicidae) focusing on the origins and use of selected strains. J Med Entomol..

[18] Rezende G, Martins A, Gentile C, Farnesi L, Pelajo-Machado M, Peixoto AA (2008). Embryonic desiccation resistance in *Aedes aegypti*: presumptive role of the chitinized serosal cuticle. BMC Dev Biol..

[19] Farnesi LC, Martins AJ, Valle D, Rezende GL (2009). Embryonic development of *Aedes aegypti* (Diptera: Culicidae): influence of different constant temperatures. Mem Inst Oswaldo Cruz..

[20] Vargas HCM, Farnesi LC, Martins AJ, Valle D, Rezende GL (2014). Serosal cuticle formation and distinct degrees of desiccation resistance in embryos of the mosquito vectors * Aedes aegypti*,* Anopheles aquasalis* and* Culex quinquefasciatus*. J Insect Physiol.

[21] Valle D, Pimenta DN, Cunha RV (2015). Dengue: teorias e práticas.

[22] Petersen LR, Jamieson DJ, Powers AM, Honein MA (2016). Zika virus. N Engl J Med.

[23] Montella IR, Martins AJ, Viana-Medeiros PF, Lima JB, Braga IA, Valle D (2007). Insecticide resistance mechanisms of Brazilian *Aedes aegypti* populations from 2001 to 2004. Am J Trop Med Hyg..

[24] Llinas GA, Seccacini E, Gardenal CN, Licastro S (2010). Current resistance status to temephos in *Aedes aegypti* from different regions of Argentina. Mem Inst Oswaldo Cruz..

[25] Achee NL, Grieco JP, Vatandoost H, Seixas G, Pinto J, Ching-Ng L (2019). Alternative strategies for mosquito-borne arbovirus control. PLoS Negl Trop Dis..

[26] Walker T, Johnson PH, Moreira LA (2011). The *w*Mel *Wolbachia* strain blocks dengue and invades caged *Aedes aegypti* populations. Nature..

[27] Pereira TN, Rocha MN, Sucupira PHF, Carvalho FD, Moreira LA (2018). *Wolbachia* significantly impacts the vector competence of *Aedes aegypti* for Mayaro virus. Sci Rep..

[28] Woke PA (1937). Comparative effects of the blood of different species of vertebrates on egg production of *Aedes aegypti*. Am J Trop Med..

[29] Paris V, Cottingham E, Ross PA, Axford JK, Hoffmann AA (2018). Effects of alternative blood sources on *Wolbachia*-infected *Aedes aegypti* females within and across generations. Insects..

[30] Christophers R (1960). *Aedes aegypti* (L): the yellow fever mosquito, its life history, bionomics and structure.

[31] Farnesi LC, Brito JM, Linss JG, Pelajo-Machado M, Valle D (2012). Physiological and morphological aspects of *Aedes aegypti* developing larvae: effects of the chitin synthesis inhibitor novaluron. PLoS One..

[32] Martins AJ, Ribeiro CD, Bellinato DF, Peixoto AA, Valle D (2012). Effect of insecticide resistance on development, longevity and reproduction of field or laboratory selected *Aedes aegypti* populations. PLoS One..

[33] McMeniman CJ, Lane RV, Cass BN, Fong AW, Sidhu M, Wang YF (2009). Stable introduction of a life-shortening* Wolbachia* infection into the mosquito *Aedes aegypti*. Science..

[34] Ant TH, Herd CS, Geoghegan V, Hoffmann AA, Steven PS (2018). The *Wolbachia* strain wAu provides highly efficient virus transmission blocking in *Aedes aegypti*. PLoS Pathogens..

[35] Shahhosseini N, Friedrich J, Moosa-Kazemi SH, Sedaghat MM, Kayedi MH, Tannich E, Schmidt-Chanasit J, Lühken R (2018). Host-feeding patterns of *Culex* mosquitoes in Iran. Parasites Vectors..

