# Correction to: The influence of different sources of blood meals on the physiology of *Aedes aegypti* harboring *Wolbachia w*Mel: mouse blood as an alternative for mosquito rearing

**DOI:** 10.1186/s13071-021-04621-9

**Published:** 2021-02-15

**Authors:** Luana Cristina Farnesi, Fabiano Duarte Carvalho, Anna Paula Canuto Lacerda, Luciano Andrade Moreira, Rafaela Vieira Bruno

**Affiliations:** 1 Laboratório de Biologia Molecular de Insetos, Instituto Oswaldo Cruz, Fiocruz, Rio de Janeiro, RJ Brazil; 2 Mosquitos Vetores: Endossimbiontes e Interação Patógeno-Vetor, Instituto René Rachou, Fiocruz, Belo Horizonte, MG Brazil; 3 Instituto Nacional de Ciência e Tecnologia em Entomologia Molecular (INCTEM)/CNPq, Rio de Janeiro, Brazil

## Correction to: Parasites Vectors (2021) 14:21https://doi.org/10.1186/s13071-020-04465-9

Following publication of the original article [1] the authors flagged that the name of one of the authors, Fabiano Duarte Carvalho, had been wrongly published as Fabiano Dias Carvalho.

The authors apologize for this mistake, which has now been corrected online and in the author list of this correction article.
